# Echoes of the Month

**Published:** 1924-08

**Authors:** 


### ECHOES OF THE MONTH.

### DR. ADAMI ON CANCER.

Dr. John George Adami, Vice-Chancellor of the University of Liverpool, at the recent annual ceremony of conferring degrees, spoke of the great progress that had attended research in connection with cancer. Greater progress was being made in the arrest and healing of cancer, he said, than had ever previously been accomplished. At the same time it was necessary, having regard to the importance of the matter to the welfare of humanity, to proceed with the utmost possible caution, so as not to encourage false hopes. We have reached the stage, however (added the Vice-Chancellor), when we can cause the disappearance of some, though not all, internal cancers.

### HOSPITAL LOTTERIES CONDEMNED.

A manifesto, signed by a number of Bishops and by the presidents of several religious bodies, has been issued condemning charity lotteries, and particularly lotteries for hospitals. The reasons given why the Churches "feel that the lottery method for hospitals and philanthropic purposes is a retrograde step" are that the appeal of the lottery is to the possibility of gain for self—an appeal directly opposed to the appeal for charity, and that the lottery method adds to the difficulty of combating the inducements to gambling, especially in young people. The Churches, nevertheless, wish to point out that they fully realise the difficulties of charitable institutions at the present time, and that they have always supported the hospitals and so feel, to some extent, responsible in respect of the methods employed by them in obtaining money.

### THE FATE OF THE ATHLETE.

Writing in the *Spectator* on "Health and Athletics," Dr. Saleeby asks: What is the fate of the athlete in after life? The question cannot be answered in one word, and the answers are probably reassuring and extremely disconcerting in different cases. A heart hypertrophied from whatever cause is a possession which may, indeed, be necessary—as when the hypertrophy compensates for a leaking or contracted valve,—but must always be a cause of concern. Is it worth while to be in your 'Varsity boat at twenty and die of the after effects of "rowing heart" in your 'forties? Or is the innuendo in that question unwarranted? We need such evidence as could be afforded by insurance companies, and by medical men of long experience who have been leading athletes in their time and have often been consulted by such. On general or *a priori* grounds I, for one, find it very hard to believe that the state of collapse sometimes witnessed at the end of the boatrace and of other races, can really be regarded with equanimity if we care to consider the duration and quality of the contests of very different kinds in which the young warrior is expected to engage in coming years.

### LOWEST AND HIGHEST.

Dr. Thomas, M.O.H. for Stepney, claims that, though a poor and congested district, it has the lowest maternal mortality in the United Kingdom—less than 2.2 per 1,000 births. "This," Dr. Thomas says, "is partly due to antenatal inspection, and to the fact that 27 per cent. of all the births occur in lying-in hospitals and other institutions, where the mothers have skilled attention." The infant mortality rate was 62 per 1,000 births—the lowest recorded in the borough—compared with 211 per 1,000 in the first year of the Borough Council's existence. On the other hand, **mortality among mothers in Kensington last year attained the highest figure recorded in the borough in any year of the present century. The actual number of deaths of mothers in Kensington last year was only seventeen, but this figure represents a death-rate of 5·4 mothers per thousand births.**

### SUN BATHS IN LONDON.

Arrangements are being completed for sun-bath facilities in Ken Wood, adjoining Hampstead Heath. The grounds are considered most suitable for these baths, which, taken in the least amount of clothing possible, are of benefit to consumptives, sufferers from bloodlessness, and people otherwise physically fit who have become run down. Near the pond at present used for mixed bathing it is proposed to erect two enclosures. In these the sexes will be segregated and patients in them may wear as little costume as they like. Should they go outside for a swim or a game of Badminton, then probably a University bathing costume will be compulsory, but of gay colour, as dark hues are bad for sunlight. The organisation will be under the auspices of the Sunlight League, and among the medical men responsible for the experiment, the first in London, will be Dr. Leonard Hill, of the National Institute of Medical Research, Dr. Saleeby, and Sir William Simpson.

### THE DEATH RATE IN FRANCE.

In an article on "The Population of France," the Paris correspondent of *The Times* writes that the prevalent practice of putting children out to nurse is believed to result in the death of 50 per cent. of those so treated. But high infant mortality alone does not wholly account for the greater death-rate in France. It is evident that the Frenchman is exposed to greater risks than the Englishman all through his life, as well as in his cradle. French statistics as to the causes of death are not available for any year later than 1918. But tuberculosis is known to be one of the chief plagues of France, and, generally speaking, the high death-rate may be assigned to ignorance and defective hygienic conditions. France is an agricultural country, whereas in England and Wales half the population lives in towns of more than 40,000 inhabitants.

### SAFETY CHART FOR OPERATIONS.

A new "safety chart" for patients under operations was announced in the Congress of Anæsthetics, held in Chicago, as the best method of avoiding the time-honoured verdict: "The operation was a success, but the patient died." The new chart records the patient's blood-pressure at five-minute intervals to show that he is always in the zone of safety. Dr. E. I. McKesson, in co-operation with his operator, Dr. C. W. Moots, after making some 10,000 records in his own work, has formulated this guiding rule: If the heart load of the patient lies between 40 and 60 per cent. the patient is operable; if outside these percentages, he is most probably inoperable. The study of 1,000 records shows that the death-rate of patients who were operable under this rule was 3 per cent., and the death-rate of the inoperable cases was over 23 per cent. There has hitherto been no sure method of diagnosing shock. These five-minute readings now enable the anæsthetist to warn the surgeon of the onset of the shock thirty minutes before it becomes apparent in any other way.

### A TYPHOID CASE IN THE WILDS.

An article on "The Call of the Wild" in "Una," the journal of the Royal Victorian Trained Nurses' Association, describes how pioneers in inland Australia may fare when illness befalls them. "About two hundred miles from the railway we pulled up at a cattle station where a girl of eighteen years lay ill: for five or six weeks she had been suffering with typhoid. There had been one and a-half inches of rain in two years, and all about the station dead beasts lay; the one-time garden was a bare sand patch, and how to diet a typhoid in that country was a problem. No fresh fruit or vegetables for 500 or 600 miles, no eggs, no milk except in tins, fresh meat once a week when the mail came along; jellies would only set occasionally during the night and had to be used in the eary morning. The salvation of the patient was a flock of young chickens, whose numbers dwindled daily, until a stronger diet was permissible. Lemons were ordered from 'down South,' but took a month to reach us. A milking goat was sent by a friendly neighbour 100 miles away, but even that failed and went 'dry.' Eventually the patient, eighteen years of age, recovered, but not before her weight dwindled to 64 lbs."

### SUMMER HEALTH SCHOOLS.

The Universities of Columbia, Iowa, Michigan and California are inaugurating summer schools for the study of the principles of public health. The object is to give a thoroughly modern training on intensive lines to anybody who wishes to undertake public health work as a vocation. Moreover, it is hoped that these schools will do their part in promoting a better system of co-operation in preventing disease by bringing into closer touch doctors, health advisers and members of the lay public. The course is to last for about seven or eight weeks, and the aim will be to concentrate as much work into that period as is consistent with efficiency. It should be said that the lay rather than the medically qualified element forms the larger proportion of the health workers in the United States, and it is estimated that the next ten years will see the entrance of thousands of citizens belonging to both sexes into various spheres of public health activities. It is to meet the needs of such persons who are without the ordinary academic—certainly without medical—training that these University summer schools have been designed.

### THE LIPSTICK DANGER.

The use of the lipstick may not be the innocuous process that is commonly thought. Recent cases of obscure dyspeptic trouble in girls have led to the suspicion that lip-sticking may be responsible. The lip salves commonly used are harmless enough, so far as digestion is concerned, if they are carefully prepared from pure ingredients. Even then the constant application, although it can do no internal harm, is damaging to the mucous membrane of the mouth, and leads to dry, cracked lips. Moreover, it results in colourlessness of the lips if the practice is temporarily suspended. When the lip salve is of a cheap variety the harm done may be more serious. In the course of the day a considerable amount may be swallowed. When the ingredients are pure this may be harmless enough. But when they are replaced by substitutes, mainly consisting of hard paraffin and some aniline dye, the perpetual swallowing of minute portions of coal-tar products must eventually result in injury to the stomach.

### CEASE FIRING!

In his Annual Report the Medical Officer of Glossop writes that the practice of firing chimneys ought no longer to be tolerated. This disgusting and dirty practice is almost universal throughout the Borough. A precise time has been fixed for it to be carried out—4.30 a.m. to 6 a.m.—and no legal action is taken if chimneys are fired in these hours. This is, of course, illegal, and not only illegal but injurious to the public health. Soot is no harmless substance; continued application of it to the skin causes cancer; continuous application of it to the delicate interior of the lungs is bound to do damage, and no less from 4.30 a.m.—6 a.m. than any other time.

### NINETY-NINE.

Sir William Hall-White, the President of the Royal Society of Medicine, has been telling the world why it is that the doctor asks us to say "ninety-nine" when he claps the stethoscope between our shoulders. "By noting how the spoken voice is carried through the chest by the stethoscope to the trained ear, a physician can obtain evidence whether the patient's chest contains air, solid or fluid. Since the words 'ninety-nine' are produced in the larynx they are most suitable for this purpose."

### VACCINATION BY THE MOUTH.

In vaccination by the mouth against typhoid, remarkable results are said to have been reported by Dr. A. Gauthier, French Surgeon-General. The vaccine was taken fasting on three successive days, and a total of nearly 4,000 vaccinations by the mouth was reached among refugees and other in Greece. A town in which there had been 200 cases of typhoid in two months developed only one new case after more than 1,000 persons had taken the vaccine. The new vaccination was less objectionable to the inhabitants than the old, and the entire population was protected, notwithstanding the fact that the drinking water—the source of the contagion—was allowed to go unpurified. In two large villages, where the population all took the vaccine, the epidemic was checked at once, only four new cases appearing. The application was very simple, and the unpleasant effects, if any, were very slight.

### SIR WILLIAM WILLCOX ON DRUG ADDICTION.

Sir William Willcox, delivering the presidential address at the annual meeting of the Society for the Study of Inebriety, said that it was the intention of the council of the society to devote increased attention to the subject of drug addiction. The problem of the limitation of the international and national supply of drugs which might give rise to addiction habits needed careful consideration. Could not some addiction drugs, such as heroin, for instance, be prohibited entirely? Were not many drugs in common use for insomnia, for example the veronal group of drugs, frequently giving rise to addiction habits? Should they not be brought within the sphere of the dangerous drugs regulations?

